# Consequences of supplying methyl donors during pregnancy on the methylome of the offspring from lactating and non-lactating dairy cattle

**DOI:** 10.1371/journal.pone.0189581

**Published:** 2017-12-11

**Authors:** Alex Bach, Anna Aris, Isabel Guasch

**Affiliations:** 1 Institució Catalana de Recerca i Estudis Avançats (ICREA), Barcelona, Spain; 2 Department of Ruminant Production, IRTA (Institut de Recerca i Tecnologia Agroalimentàries), Caldes de Montbui, Spain; 3 Blanca, Hostalets de Tost, Spain; University of Missouri Columbia, UNITED STATES

## Abstract

The aim of this study was to evaluate the potential effects of methyl donor supplementation of pregnant animals in the presence or absence of a concomitant lactation on the methylome of the offspring. Twenty Holstein cows, 10 nulliparous (non-lactating while pregnant) and 10 multiparous (lactating while pregnant) were blocked by parity and randomly assigned to an i.m. weekly injections of a placebo (**CTRL**) or a solution containing methyl donors (**MET**). After calving, 5 calves randomly selected from each treatment (two born to non-lactating and three to lactating dams) were blood-sampled to determine their full methylome. There were more than 2,000 CpG differentially methylated between calves born to CTRL and those born to MET, and also between calves born to lactating and non-lactating dams. Most of the differences affected genes involved in immune function, cell growth regulation and differentiation, kinase activity, and ion channeling. We conclude that the coexistence of pregnancy and lactation affects the methylome of the offspring, and that supplementation of methyl donors early in gestation has also consequences on the methylome.

## Introduction

Despite major advances in interpreting the methylome of mammals, much is left to unfold the potential interactions between nutrition and methylation of DNA. Nutrition of the pregnant dam is an important factor exerting epigenetic regulations of the offspring. In adult dairy cows, close to 70% of the gestation coincides with lactation, and the placenta must compete for nutrients with the mammary gland [1]. However, macro-nutrient (mainly energy and protein) needs of dairy cows for pregnancy are typically ignored (and thus unknown) until cows are 190 d pregnant [2]. Furthermore, all nutritional models used in dairy cattle assume that supply of vitamin B_12_ and folic acid from rumen microbial growth is more than adequate to meet the expected requirements of lactating dairy cows [3]. González-Recio *et al*. [4] described a clear trans-generational effect on milk production, health, and longevity when pregnancy and lactation coincided, precluding the offspring born to the most productive cows to fully express their potential additive genetic merit during their adult life. It is likely that these effects were in part due to some changes in the methylome of the offspring as a consequence of nutrient competition between the placenta and the mammary gland. Yunta *et al*. [5] attempted to revert these changes by supplementing pregnant cattle with arginine to improve placental blood flow and thus provide more nutrients to the fetus; however, placental blood flow did not increase.

Folic acid and vitamin B_12_ are methyl donor compounds essential for the synthesis of DNA, RNA, and proteins, and participate in several pathways for energy production. In non-ruminant species, folic acid and/or vitamin B_12_ supplementation around conception reduces the incidence of neural tube defects [6]. Although the exact mechanisms involved in this prevention are unknown, it is likely that epigenetic mechanisms are, at least, partially responsible of this outcome. For instance, methylation of DNA occurs via de methyl donor S-adenosulmethionine (SAM), and maintenance of adequate SAM circulating concentrations are, in part, dependent on vitamin B_12_ and folic acid [7]. Lillycrop *et al*. [8] reported that a methyl donor deficiency (mainly due to lack of methionine) in pregnant rats caused epigenetic modifications on hepatic genes in the offspring, but when rats were supplemented with folic acid, these modifications did not occur. Similarly, Sinclair *et al*. [9] described widespread differences in DNA methylation between lambs either born to ewes unsupplemented or supplemented with vitamin B_12_ and folic acid. However, weekly intramuscular injections of 160 mg of folic acid starting 45 d after conception had no effect on calf birth weight and some blood parameters in dairy cattle [10].

We hypothesized that supplementation of methyl donors may alter the methylome of the offspring, especially when pregnancy coincides with lactation. Thus, the objective of this study is to use the dairy cow as a model to evaluating the potential effects of methyl donor supplementation of pregnant animals in the presence or absence of a concomitant lactation on the methylome of the offspring. However, despite the fact that several studies have reported that high degrees of CpG methylation at the promoter regions are incompatible with gene expression [11], comparative analysis using cancer cell lines [12] have shown that differences in DNA methylation elicit only small changes in gene expression. Therefore, we aimed simply at evaluating potential changes in methylation patterns of the offspring and propose or identify some potential changes in functionality, as it is probable that, unlike single gene mutations, differences in the degree of methylation at a single locus could potentially elicit a myriad of trait phenotypes [13].

## Methods

No statistical methods were used to predetermine sample size. All procedures and handling of animals in this study were performed under the supervision and approval of the Animal Care Committee of Institut de Recerca i Tecnologia Agroalimentàries (IRTA) in compliance with the European Directive 2010/63/EU. The approval number was A3002-A5018.

### Animals and treatments

Twenty Holstein cows, 10 non-lactating nulliparous (612±46 kg of body weight; average±SD) and 10 lactating multiparous (677±58 kg of body weight; average±SD) cows, once confirmed pregnant (35.7 ± 3.86 d pregnant; average±SD), were blocked by parity and randomly assigned to either an i.m. weekly injection of saline solution as a placebo (**CTRL**) or an i.m. weekly injection of a solution containing methyl donors (**MET**). Heifers (nulliparous cows non-lactating while pregnant) received a weekly i.m. injection of 11.5 mL of a solution supplying 10 mg (16 μg/kg of body weight) of vitamin B_12_ and 100 mg of folic acid (160 μg/kg of body weight) or an i.m. injection of 11.5 mL of saline solution as a placebo. Multiparous cows (lactating while pregnant) received a weekly i.m. injection of 23 mL of a solution supplying a total of 20 mg (30 μg/kg of body weight) of vitamin B_12_ and 200 mg (300 μg/kg of body weight) of folic acid or an i.m. injection of 23 mL of saline solution as a placebo. All injections took place between pregnancy diagnosis (~35 d of gestation) until cows reached 120 d of pregnancy, which involved an average (±SD) of 12.5±0.61 injections per animal during the entire study. Methyl donors were supplemented i.m. to avoid destruction of vitamins by ruminal microorganisms. The doses of methyl donor were based on previous literature [14, 15], and lactating cows received a greater dose (relative to body weight) than heifers because they have additional requirements to sustain lactation [2]. Lactating cows, both supplemented and those unsupplemented with methyl donors, received a ration that provided 1.62 Mcal of net energy of lactation/kg, 15% crude protein (and as it is typically the case, had no added folic acid or vitamin B_12_). Heifers (non-lactating), both supplemented and those unsupplemented with methyl donors, were fed a diet containing 1.42 Mcal of net energy of lactation/kg, 13% crude protein, and no added folic acid or vitamin B_12_. Between 220 d of pregnancy and calving both lactating and non-lactating cows received the same diet providing 1.35 Mcal of net energy, 12.8% crude protein, and no added vitamin B_12_ and folic acid. All animals were managed under the same conditions throughout the study. After calving, 5 female calves randomly selected from each treatment (2 born to non-lactating and 3 born to lactating dams) were blood-sampled into sodium-heparinized Vacutainer tubes within the first 6 h of life and blood was immediately transported to the laboratory to isolate peripheral blood mononuclear cells (PBMC), describing no differences in the number of PBMC isolated between calves born to primiparous or multiparous dams.

### Determination of DNA methylation

DNA was extracted from isolated PBMC with DNeasy Blood & Tissue Kit (Qiagen, Madrid, Spain) and submitted to a quality control test using a Nanodrop-1000 (JH Bio, USA) and 0.8% agarose gel to determine the purity and quantity. Then, to identify genes that could be regulated by DNA methylation upon supplementation of methyl donors, a genome-wide Methyl-MiniSeq (Zymo (Research Corporation, Irvine, CA) was performed. The sequencing resulted in >92% coverage of all CpG islands and >90% of all gene promoters was performed (defining coverage as the average number of reads that aligned to known reference bases). First, libraries were prepared from 200–500 ng of genomic DNA digested with 60 units of TaqαI and 30 units of MspI (NEB) sequentially and then extracted with Zymo Research (ZR) DNA Clean & ConcentratorTM-5 kit (Cat#: D4003). Fragments were ligated to pre-annealed adapters containing 5’-methyl-cytosine instead of cytosine according to Illumina’s specified guidelines (http://www.illumina.com). Adaptor-ligated fragments of 150–250 bp and 250–350 bp in size were recovered from a 2.5% NuSieve 1:1 agarose gel (ZymocleanTM Gel DNA Recovery Kit, ZR Cat#: D4001). The fragments were then bisulfite-treated using the EZ DNA Methylation-LightningTM Kit (ZR, Cat#: D5020). Preparative-scale PCR was performed and the resulting products were purified (DNA Clean & ConcentratorTM—ZR, Cat#D4005) for sequencing on an Illumina HiSeq. Reads were quality-controlled with FastQC (Babraham informatics, Cambridge, UK) (and trimmed to remove adapter sequences with Trim Galore (Babraham informatics, Cambridge, UK). Then, processed reads (were mapped to the *in silico* bisulfite-converted reference genome (Bovine assembly UMD3.1.1/bosTau8) (using Bismark software (version 0.16.1) [16].)

### Bioinformatics and statistical analyses

Only CpGs with a sequencing depth of >10x and a methylation difference >25% in all samples were considered for further analysis. These constraints resulted in a mapping efficiency ranging between 31 and 37%, which represented between 6,645,913 and 6,885,848 unique CpGs sites per biological replicate. The methylation level of each sampled cytosine was estimated as the number of reads reporting a cytosine, divided by the total number of reads reporting a cytosine or thymine. To identify genes, which were potentially regulated by DNA methylation changes, a differentially methylated analysis of CpG at the promoter and within the gene region was performed using Fisher’s exact test or t-test on each CpG site which had at least five reads coverage, and promoter, gene body, and CpG island annotations were added for each CpG included in the comparison. To further minimize the risk of a type II error, only significant differentially methylated sites with a methylation change >30% and a p-value < 0.01 were used for further analysis. All statistical analyses, clustering, and generation of heatmaps was performed with the software package ‘R’ [17] with the methylKit library [18]. Last, all genes with a significant (p < 0.01) DNA methylation change of >30% were subjected to DAVID Gene Functional Clustering tool (Database for Annotation, Visualization and Integrated Discovery) (http://david.abcc.ncifcrf.gov) to classify the list of genes differentially methylated within biological processes using *Bos taurus*(7) as reference species.

## Results

### Effects of absence or presence of lactation during pregnancy on the methylome of the offspring

**Fig 1:**
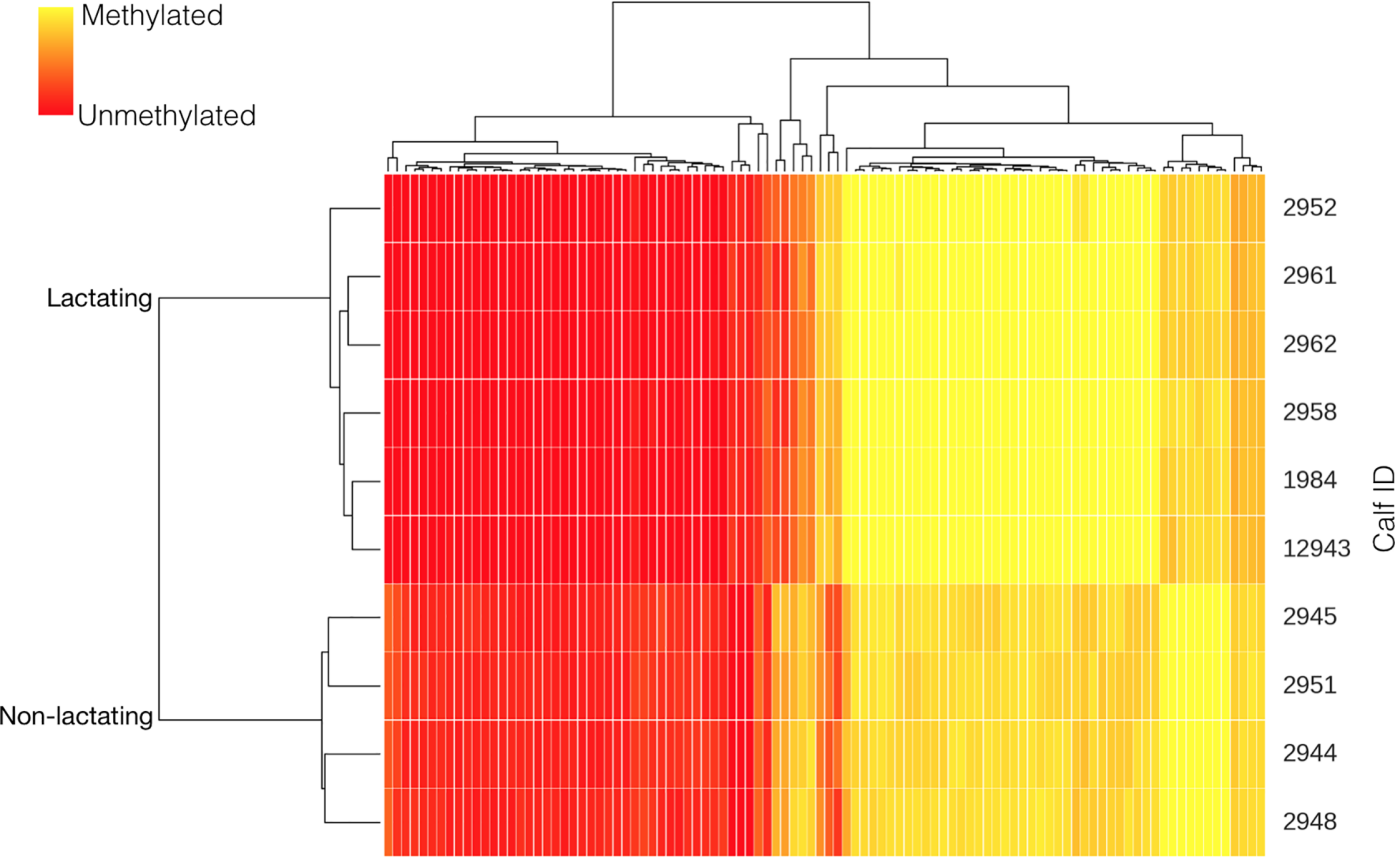
Cluster analysis of CpG sites differentially methylated (p < 0.01) in the offspring as affected by absence or presence of lactation during gestation.

The potential effect of lactation during pregnancy on the methylome of the offspring was assessed using 5 replicates per treatment. There were no differences (p = 0.15) between global methylation level in calves born to non-lactating heifers (63±3.95%) or born to lactating dams (54±3.22%). However, there were clear differences in the methylation pattern between the offspring born to non-lactating and those born to lactating cows (Fig 1). There were 11 CpG at the promoter region (*VSIG3*, *RAB7B*, *ENG*, *TNSF15*, *GPS2*, *TNFSF15*, *PCDH17*, *UCN*, *KBTBD6*, *IFF01*, and *PCDH17*) and 112 CpG at the gene region (both intron and exons) that were hypermethylated (>30% differentially methylated; p < 0.01) in calves born to non-lactating heifers compared with calves born to lactating cows. Contrarily, there were 14 CpG at the promoter region (*CDX1*, *ECE1*, *FXYD2*, *ZFP64*, *MIR2896*, *PARVB*, *SAMD5*, *FXYD2*, *ZFP64*, *FXYD2*, *CALY*, *RUNX3*, and *BATF*) and 71 CpG at the gene region (both intron and exons) that were hypomethylated (>30% differentially methylated; p < 0.01) in calves born to non-lactating heifers compared with calves born to lactating cows.

The genes that were hypermethylated at the promoter region in calves born to non-lactating heifers compared with calves born to lactating cows could be grouped into three functional clusters using DAVID. These functions pertained to immune response, chromatin structure, and the nervous central system. The genes that were hypomethylated at the promoter region could not be grouped into any cluster.

The 112 CpG that were hypermethylated in calves born to non-lactating heifers compared with calves born to lactating cows at the gene region in calves born to heifers compared with calves born to multiparous cows could be grouped, according to DAVID, into 1 large functional cluster related to immune function (i.e., *PCDHGA8*, *PCDHGB4*, *VSIG2*); whereas the 71 genes that were hypomethylated at the gene level could not be grouped into specific functional clusters.

### Methyl donor supplementation and methylome of the offspring

**Fig 2:**
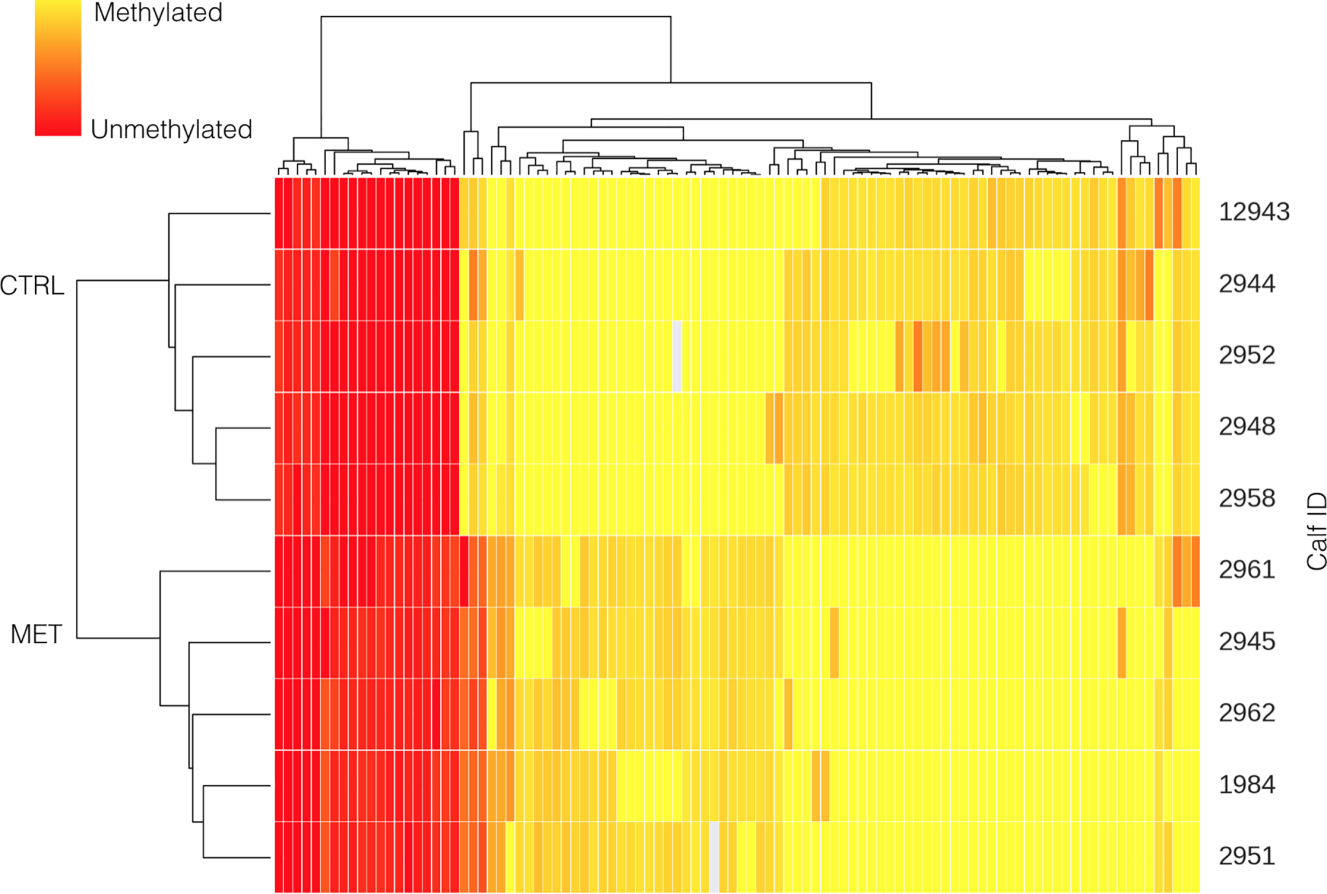
Cluster analysis of CpG sites differentially methylated (p < 0.01) in the offspring as affected by supplementation of methyl donors of their dams during early pregnancy. Control dams received a placebo, whereas lactating MET dams received weekly administrations of 200 mg of folic acid and 20 mg of vitamin B_12_ and non-lactating MET dams received weekly supplementation of 100 mg of folic acid and 10 mg of vitamin B_12_.

Again, the potential effect methyl donor supplementation during early pregnancy on the methylome of the offspring was assessed using 5 replicates per treatment. There were no differences (p = 0.66) between global methylation levels in calves born to unsupplemented (58.8±2.55%) or supplemented dams (57.2±2.55%). However, there were more than 2,000 CpG sites that were differentially (p < 0.001) methylated between calves born to CTRL and calves born to MET dams (Fig 2). Within these CpG that were strongly differently methylated, 32 CpG at the promoter regions (>30% differentially methylated; p < 0.01) and 587 CpG sites at the gene region (>30% differentially methylated; p < 0.01) were hypermethylated. Likewise, 27 and 437 CpG sites at the promoter and gene regions, respectively were heavily hypomethylated (>30% differentially methylated; p < 0.01).

Using DAVID, 4 genes (*CEND1*, *VSIG2*, *B3GNT8*, *FAM69A*) that were hypermethylated (>30% differentially methylated; p < 0.01) in calves born to CTRL compared with calves born to MET dams at the promoter region were related to immune function, cell differentiation, and neural differentiation, but the rest of genes could not be clustered by function, but there were some genes previously described as imprinted genes such as *IGF2*. Also, 4 CpG were hypomethylated (>30% differentially methylated; p < 0.01) at the promoter region (*AVPR1B*, *BDKRB2*, *ADRA1D*, *PTGER3*) in calves born to CTRL compared with calves born to MET dams and could be grouped in 1 cluster related to calcium metabolism and vascular function. Within the group of hypermethylated CpG at the gene region, the three most important functions were related to immune function (e.g., *boLA*, *MIC2*, *IGLL1*, *MIC1*), regulation of cell growth and differentiation (e.g. *TBC1D14*, *TBC1D5*, *TBC1D16)*, and kinase activity (e.g, *PRKD3*, *SGK2*, *DCLK1*, *RPS6KA2*). Within the group of hypomethylated genes at the gene region, the three most important functions were related to cell signaling (e.g., *SIK2*, *MAPK2K5*, *RAF1*, *MARK2*), membrane proteins (e.g., *GPR37L1*, *DP6*, *CCKBR*, *APMAP*), and zinc-finger proteins (e.g., *ZNF296*, *HIC2*, *ZIC3*, *ZNF423*).

### Methyl donor supplementation of lactating dams and methylome of the offspring

**Fig 3:**
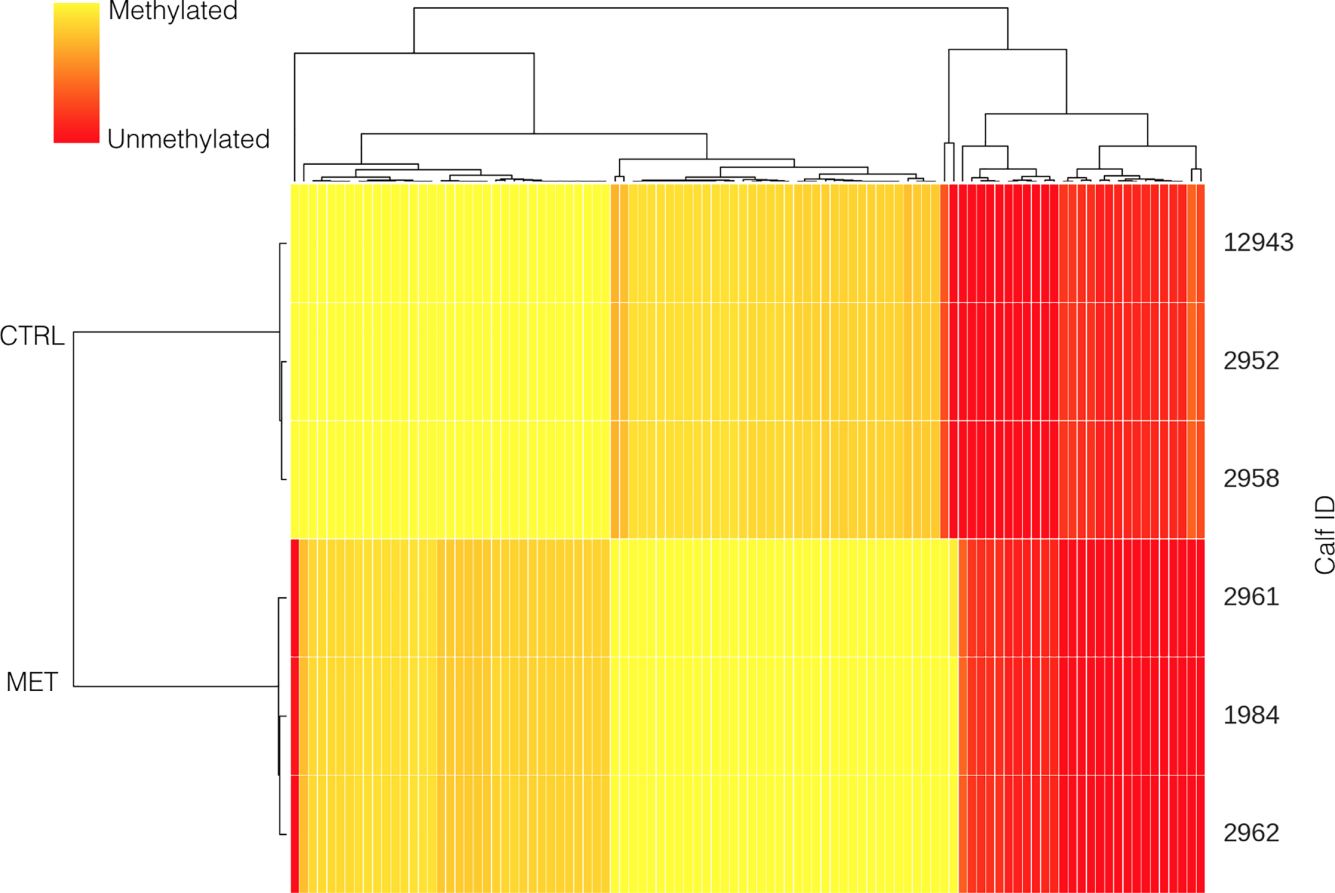
Cluster analysis of CpG sites differentially methylated (p < 0.01) in the offspring born to lactating dams that received a supplementation of methyl donors or a placebo during early pregnancy. Control dams received a placebo, whereas MET dams received weekly administrations of 200 mg of folic acid and 20 mg of vitamin B_12_.

The potential effect methyl donor supplementation during early pregnancy in lactating dams on the methylome of the offspring was assessed using 3 replicates per treatment. Supplementing pregnant and lactating cows with methyl donors had no effect (p = 0.54) on overall methylation level (54.6±3.22%) compared to calves born to unsupplemented lactating cows (55.3±3.22%). However, methyl donor supplementation during early pregnancy of lactating cows resulted in hypermethylation (>30% differentially methylated; p < 0.01) of 30 CpG sites in the promoter region and 161 CpGs in the gene region, and hypomethylation (>30% differentially methylated; p < 0.01) of 34 CpG in the promoter region and 157 CpG sites in the gene region compared with the offspring from CTRL heifers (Fig 3).

None of the CpG strongly hypermethylated or hypomethylated in the promoter could be clustered using DAVID. However, 9 of the 161 genes that were strongly hypermethylated at the gene region in lactating dams supplemented with methyl donors during early pregnancy could be clustered into 2 groups related to gene expression and neural function (e.g., *NSG1*, *SORCS3*, *TMEM145*, *ENTPD8*, *PTCRA*, *SLC6A9*). The 157 genes that were strongly hypomethylated at the gene region could not be clustered by functionality either.

### Methyl donor supplementation of pregnant and non-lactating dams and methylome of the offspring

**Fig 4:**
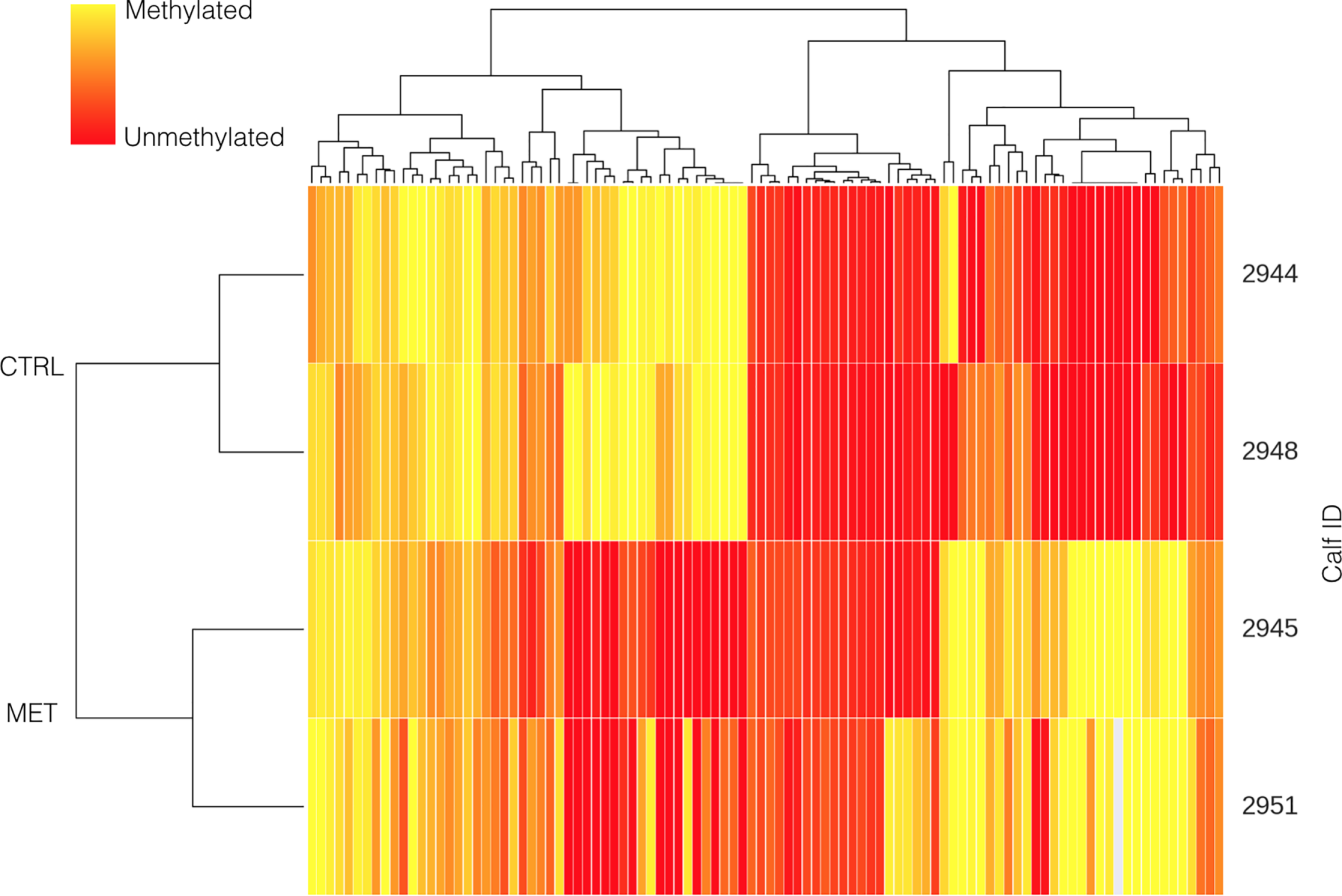
Cluster analysis of methylation status of CpG sites differentially methylated (p < 0.01) in the offspring born to non-lactating dams that received a supplementation of methyl donors or a placebo during early pregnancy (while lactating). Control dams received a placebo, whereas MET dams received weekly administrations of 100 mg of folic acid and 10 mg of vitamin B_12_.

The potential effect methyl donor supplementation during early pregnancy in non-lactating dams on the methylome of the offspring was assessed using 2 replicates per treatment. There were no differences (p = 0.54) between global methylation in calves born to unsupplemented non-lactating dams (63.0±4.0%) or born to supplemented non-lactating dams (59.0±4.0%). Supplementing non-lactating cows with methyl donors during early pregnancy resulted in hypermethylation (>30% differentially methylated; p < 0.01) of 63 CpG sites in the promoter region and 464 CpG in the gene region, and hypomethylation (>30% differentially methylated; p < 0.01) of 47 CpG in the promoter region and 438 CpG sites at the gene region of the offspring (Fig 4).

Among the CpG sites at the promoter region that were strongly hypermethylated in non-lactating cows supplemented with methyl donors during early pregnancy, there was a strong cluster related to immune function and in particular to major histocompatibility complex through the gene *BOLA*, whereas CpG sites at the promoter region that were strongly hypomethylated could not be grouped into specific clusters, although they were mainly involved in the regulation of gene expression (e.g., *MIR2887*, *MIR631*, *MIR2904*) and neural function (e.g., *MOG*, *CHRM1*) among other activities.

Similar to what was observed when comparing supplemented and unsupplemented dams that were pregnant while lactating, DAVID made 10 clusters grouping the 467 CpG sites strongly hypermethylated at the gene region. The three most important clusters were related with immune function (e.g., *BOLA*, *IGLL1*, *CECAM19*), cell signaling (e.g., T*BC1D14*, *TBC1D5*, *TBCD16*), and ion channeling (e.g., *KCNC4*, *KCTD5*, *KCNU1*, *KCNS1*). Last, among the 438 CpG sites strongly hypomethylated at the gene region, DAVID grouped them into 5 clusters, with the three most relevant pertaining to cell signaling (e.g., *MAP2K5*, *RAF1*, *MARK2*), cell membrane proteins (e.g., *APMAP*, *DPP6*, *GPR37L1*, *SLC6A6*), and zinc-finger proteins (e.g., *ZIC3*, *HIC2*, *ZNF75D*).

## Discussion

The results presented herein illustrate substantial changes in the methylation pattern of the offspring born to dams that were or were not lactating while pregnant. To our knowledge, this is the first study that shows an epigenetic effect of lactation during pregnancy. There are several studies that show that nutrient restrictions of small deficiencies of micronutrients may lead to alterations in the methylome of the offspring [19], and it is likely that the co-existence of pregnancy and lactation may elicit some nutrient shortages at placental or fetal level leading to differences in the methylation degree of selected genes in the offspring. However, by the design, and due to the biological development and cycle of cattle, the potential effect of parity is confounded herein with the potential effect of lactation, because non-lactating cows were about 12 months younger than lactating cows.

The lack of changes in global methylation degrees among treatments is in accordance with previous studies that have evaluated the effects of methyl donor supplementation on the methylome [9]. However, in the current study, supplementation of methyl donors during pregnancy resulted in hypermethylation of CpG at the promoter region of genes related to immune function, cell differentiation, and neural differentiation, and hypomethylation of CpG at the promoter region of genes related to vascular function. In both, humans [20, 21] and rats [22] maternal vitamin B_12_ deficiency is associated with reduced birth weight and increased risk of insulin resistance. Also, Steegers-Theunissen *et al*. [23] reported changes in the methylation status of the gene *IGF2* when comparing 17-month old babies born to mothers supplemented or not with folic acid periconceptionally, and described an association between birth weight and methylation status of *IGF2*. Interestingly, in the current study calves born to dams that received methyl donors had the CpG at the gene region of *IGF2* hypermethylated (differentially methylated >30%; p < 0.01) compared with those born to unsupplemented dams.

There were also important differences in the methylation pattern (Fig 3) between calves born to supplemented and unsupplemented lactating dams, with most of these differences affecting genes involved in gene expression mechanisms and neural function. These differences were also evident (Fig 4) in calves born to non-lactating cows, even with the limited sample size used for this comparison (*n* = 2). In this case, important differences in methylation status were observed in genes related also to immune function, gene expression, ion challenging, and cell signaling at the promoter region in calves born to non-lactating cows in the MET treatment compared with those born to non-lactating cows in the CTRL group.

These functional clusters were similar to those reported by Gearghty *et al*. [19] who evaluated changes in the blood methylome of adult healthy females that were unsupplemented or supplemented with methyl donors. The large number of CpG differently methylated in calves born to lactating cows not supplemented compared with those that were born to lactating cows that were supplemented during early pregnancy suggests that the co-existence of gestation with lactation exerts an increased demand of methyl donors, and thus providing these supplements seems more important in lactating than in non-lactating cows. Milk production requires substantial amounts of glucose for the synthesis of lactose. Propionate, which is a major rumen fermentation end-product, is the main source of glucose in dairy cows. Propionate enters the Krebs cycle and gluconeogenesis by the action and the pathway requires the participation of methylmalonyl CoA mutase, which is a vitamin B_12_-dependent enzyme. There is evidence that supplementation of folic acid and vitamin B_12_ increases the expression of the gene coding for methylmalonyl-CoA mutase in dairy cattle [14] and also increases milk production during early lactation [24]. On the other hand, folic acid is part of coenzymes that participate in the transfer of one-carbon unit in several biochemical pathways. Milk production during the first 200 d of lactation was increased linearly in multiparous, but not in primiparous cows fed a diet that provided 0, 2, or 4 mg of folate/kg of body weight [25], and an intramuscular administration of folic acid (320 mg/week) and vitamin B_12_ (10 mg/week) about 1 month before and 2 months after calving reduced time to first breeding [26]. Gagnon *et al*. [15] reported that an intramuscular injection of folic acid and vitamin B_12_ during 24 d before and 56 d after calving had minor consequences on milk yield, but they reported a number of changes in the expression of several genes in the granulosa cells, and provided some support to the hypothesis that lactating cows may not be able to meet their needs of methyl donors through conventional rations. More recently, Duplessis *et al*. [27] have reported changes in energy partitioning in early lactation cows that were supplemented with methyl donors, which would indicate that, in fact, availability of methyl donors during lactation may be limited.

## Conclusions

This is the first study showing a wide epigenetic effect of the overlap of pregnancy and gestation. It also shows that supplementation of methyl donors during pregnancy, both in the presence and absence of lactation, in cattle exerts modification in the methylation status of several genes in the offspring, with these changes being more marked in calves bon to dams that were lactating while pregnant than in those that were not lactating. Further research is needed, though, to evaluate the actual consequences of these differences in the methylome.

## Supporting information

S1 Table Top 2000 CpG sites differentially methylated in the offspring as affected by absence or presence of lactation during gestation. (XLS)

S2 Table Top 2000 CpG sites differentially methylated in the offspring as affected by supplementation of methyl donors of their dams during early pregnancy. (XLS)

S3 Table Top 2000 CpG sites differentially methylated in the offspring born to lactating dams that received a supplementation of methyl donors or a placebo during early pregnancy. (XLS)

S4 Table Top 2000 CpG sites differently methylated in the offspring born to non-lactating dams that received a supplementation of methyl donors or a placebo during early pregnancy. (XLS)
